# Soy Protein Concentrate Dietary Substitution and *Bacillus coagulans* Supplementation Influence the Growth Performance, Digestive Enzyme Activity, Antioxidant Capacity, Gene Expression, and Gut Microbiota of Hybrid Grouper (*Epinephelus fuscoguttatus*♀ × *Epinephelus lanceolatus*♂)

**DOI:** 10.1155/anu/4176337

**Published:** 2026-04-22

**Authors:** Yirong Yuan, Zhen Zhang, Danlu Sun, Qi Liu, Li Wang, Yonggan Chen

**Affiliations:** ^1^ Key Laboratory of Utilization and Conservation for Tropical Marine Bioresources (Hainan Tropical Ocean University), Ministry of Education, Sanya, China, meb.gov.tr; ^2^ Yazhou Bay Innovation Institute, Hainan Tropical Ocean University, Sanya, China, qzu.edu.cn; ^3^ College of Fisheries and Life Science, Hainan Tropical Ocean University, Sanya, China, qzu.edu.cn

**Keywords:** *Bacillus coagulans*, hybrid grouper, soy protein concentrate

## Abstract

This study evaluated the effects of soy protein concentrate (SPC) substitution for fish meal and dietary supplementation of *Bacillus coagulans* on the growth performance and biochemical responses of juvenile hybrid grouper (*Epinephelus fuscoguttatus*♀ × *Epinephelus lanceolatus*♂). Three experimental diets were designed, including 0% SPC substituting fish meal (NSPC0), 25% SPC substituting fish meal (NSPC25), and 25% SPC substituting fish meal supplemented *B. coagulans* to 10^8^ CFU/g (BSPC25). As a result, BSPC25 significantly improved the final body weight (FBW), specific growth rate (SGR), weight gain rate (WGR), and condition factor (CF). Compared with NSPC0, BSPC25 significantly elevated the liver expression level of rapamycin target (TOR) gene, and NSPC25 and BSPC25 notably increased the content of malondialdehyde (MDA) in the liver. However, there was no significant difference in superoxide dismutase (SOD) activity, gut microbiota α‐diversity, and β‐diversity. In summary, 25% SPC substitution of fish meal together with *B. coagulans* dietary supplementation can significantly improve the fish growth performance by upregulating the TOR gene expression, providing important insights into the development of low‐fish meal diets for hybrid grouper.

## 1. Introduction

The shortage of fish meal resources has become increasingly prominent with the continuous development of aquaculture, and the development of plant protein sources to substitute fish meal has become a research hotspot [1]. Soybean is often used as a plant protein additive in feed due to its high protein content and relatively balanced amino acid composition [2]. Soy protein concentrate (SPC) is a purified protein derived from soybeans via a low temperature desolvation process, which can retain more crude protein than soybean meal while removing soluble carbohydrates, ash, and other substances [3, 4]. The growth performance of most aquatic animals can be maintained under a high proportion of soybean substitution for fish meal; however, the anti‐nutritional factors, such as oligosaccharides, lectins, allergens, trypsin inhibitors, may inhibit digestion and absorption and cause intestinal inflammation in fish [5, 6]. These negative effects largely limit a further increase in the proportion of soybean protein substitution.

To address the aforementioned limitations, probiotics supplementation has been increasingly applied in aquaculture due to its high safety, environmental friendliness, and multifunctional regulatory effects on the physiological metabolism of fish [7, 8]. Some probiotics can secrete digestive enzymes to decompose proteins. *B. coagulans* has been widely applied to livestock and poultry production, exhibiting significant effects to improve the growth performance, microbial composition, and immune function [9–11]. Previous studies have demonstrated that *B. coagulans* could improve fish growth performance, intestinal microbial diversity, and resistance to oxidative stress [12, 13].

Hybrid grouper has become a popular farmed fish species with high nutritional value in recent years, which combines the advantages of fast growth of *E. lanceolatus* and strong disease resistance of *E. fuscoguttatus*. As a carnivorous fish species, it has high requirements for dietary protein. To overcome the limitations of soybean protein in substituting fish meal [14], some studies have attempted to supplemented low‐fish‐meal diets with *B. coagulans*, which was found to improve the digestive enzyme activity, intestinal barrier function, and immune function of *Macrobrachium rosenbergii* [15]. Therefore, this study supplemented *B. coagulans* to SPC‐substituted fish meal feeds and observed its effects on the growth performance, digestive enzyme activity, antioxidant enzyme activity, gene expression, and gut microbiota of juvenile hybrid grouper. The findings may provide important insights into the development of low‐fish meal diets for grouper.

## 2. Materials and Methods

### 2.1. Diet Ingredients and Experimental Strains

The diets were formulated with fish meal, casein, shrimp meal, and wheat flour as the main protein sources, and SPC was added to substituted 0% (NSPC0) and 25% (NSPC25) of fish meal, respectively. Soybean oil and fish oil served as the primary lipid sources, and various trace elements were also supplemented (Table 1). The raw materials were thoroughly mixed, processed into pellets with a diameter of approximately 1–2 mm, dried, and then sealed and stored at 4°C. *B. coagulans* Y35 was isolated from *Epinephelus* in our laboratory. The strain solution was prepared using LB medium at 45°C, under shaking at 200 rpm, incubation for 24 h, centrifugal washing for 3 times, sealed and stored at 4°C for use. The strain solution was resuspended with normal saline and mixed with NSPC25 to prepare a pellet, dried at 45°C for 24 h, with the final concentration of *B. coagulans* to 10^8^ CFU/g, which was the BSPC25 group. Sealed and stored at 4°C for use.

**Table 1:** Ingredient composition and nutrient content of the tested diets (% dry matter).

Ingredients	NSPC0	NSPC25
Fish meal^a^	65.0	48.75
Soy protein concentrate^b^	0.0	15
Casein	5.0	5.0
Shrimp meal	2.5	2.5
Flour	18.0	17.6
Binder	2.5	2.5
Soybean oil	1.0	1.825
Fish oil	1.0	1.825
Squid viscera paste	1.5	1.5
Vitamin premix^c^	1.0	1.0
Mineral premix^d^	1.0	1.0
Choline chloride	0.5	0.5
Dicalcium phosphate	1.0	1.0
Analyzed composition		
Crude protein (%)	45.89	45.84
Crude lipid (%)	10.40	10.08
Dry matter (%)	90.33	90.51
Ash (%)	12.09	10.36
NFE	21.95	24.23

^a^Fish meal: crude protein 60%, crude lipid 12%, dry matter 18%, moisture 10%, lysine 3%, and methionine 1.5%.

^b^Soy protein concentrate: crude protein 65%, dry matter 7%, and moisture 10%.

^c^Vitamin mixture (g/kg mixture): lysine, 10 g; vitamin A, 0.03 g; vitamin C, 30 g; vitamin B1, 0.1 g; vitamin D3, 0.0025 g; vitamin E, 0.0045 g; and nicotinic acid, 0.1 g. All ingredients were diluted with corn starch to 1 kg.

^d^Mineral premix (g/kg mixture): Cu, 12 g; Zn, 32 g; Fe, 20 g; Mn, 40 g; I, 0.8 g; and Se, 0.4 g. All ingredients were diluted with corn starch to 1 kg.

### 2.2. Experimental Fish and Experimental Design

The experimental fish was juvenile hybrid grouper purchased from a commercial farm (Ledong, China). Acclimation was carried out for 14 days before the experiment. After adaptation, the fish (initial body weight: 8.10 ± 0.04 g) were randomly divided into three groups (each consisting of 15 fish) with three replicates, which were fed with NSPC0, NSPC25, and BSPC25, respectively. The feeding trials were carried out with circulating water and oxygen equipment, and the temperature was maintained at 27 ± 2°C. The feeding amount was adjusted according to the total weight of the fish in each experimental unit, and feeding was carried out twice a day at 9:00 and 18:00. The residual impurities were cleaned up after feeding and the growth experiments were conducted for 60 days.

### 2.3. Sample Collection

After the fish had fasted for 24 h, the samples were collected. Seven fish are randomly collected per box. Under sterile conditions, dissection was performed using a sterilized scalpel, and the intestines, intestinal contents, and liver were taken and stored in dry ice for quick freezing. The intestinal contents were scraped out with sterile forceps for microbiota analysis. The foregut was taken for digestive enzyme activity analysis, and the midgut was used for PepT1 expression analysis. The liver was used to analyze the antioxidant enzyme activity and IGF‐1, GH, and TOR expressions.

### 2.4. Detection of Intestinal Digestive Enzymes

The biochemical assay kit of Solarbio Technology Co., Ltd. (Beijing, China) was used to detect trypsin (Solarbio trypsin Activity Detection Kit, BC2315), amylase (Solarbio α‐Amylase Activity Detection Kit, BC0615), and lipase (Solarbio lipase Activity Detection Kit, BC2345) activities. According to the operation steps, 0.1 g of tissue sample was taken and added to the extracting solution at a 1:5–1:10 ratios, ground evenly, and centrifuged at 4°C for 10 min to obtain the supernatant for further analysis.

### 2.5. Determination of Antioxidant Enzyme Activities

The antioxidant enzyme activity detection kit was provided by Solarbio Technology Co., Ltd. (Beijing, China). The activities of superoxide dismutase (SOD; Solarbio SOD Activity Detection Kit, BC5165) and malondialdehyde (MDA; Solarbio MDA Activity Detection Kit, BC0025) in the liver were detected according to the guidance of the kit by the trace method.

### 2.6. RT‐qPCR

Total RNA was isolated from the intestinal and liver tissues using Trizol reagent (Invitrogen). RNA integrity was checked using a Nano Drop 2000 spectrophotometer (Thermo Scientific, USA). High‐purity RNA was used for cDNA synthesis, and RT‐qPCR analysis was used to determine the expression levels of related genes, including PepT1, IGF‐1, GH, and TOR. The reaction mixture consisted of 1 μL of cDNA, 0.4 μL of forward primers, 0.4 μL of reverse primers, 10 μL 2 × SYBR real‐time PCR premixture, and 8.2 μL RNase‐free ddH_2_O. The β‐actin gene was selected as the reference gene‐specific primer for RT‐qPCR, and detailed information about the primers can be found in Table 2 (Supporting Information).

**Table 2:** Nucleotide sequences of the primers used in RT‐qPCR.

Genes	Primer sequence (5′–3′)	GenBank ID
PepT1^a^	F^e^: CCATCAATGCTGGCAGTCTA R^f^: CCACTTCCAACAATGAACACAA	JX122768.1
IGF‐1^b^	F: TATTTCAGTAAACCAACAGGCTATG R: TGAATGACTATGTCCAGGTAAAGG	AY776159.1
GH^c^	F: AGAGACTCTTCTCCGACTTTGAG R: CTCCCAGGACTCCACCAAC	EU280321.1
TOR^d^	F: TCTCCCTGTCCAGAGGCAATAA R: CAGTCAGCGGGTAGATCAAAGC	JN850959.1
β‐actin	F: AAGATGAAATCGCCGCAC R: GCTCCTCAGGGGCAACTC	KU200949.2

^a^Oligopeptide transporter 1 (PepT1).

^b^Insulin growth factor 1 (IGF‐1).

^c^Growth hormone (GH).

^d^Target of rapamycin (TOR).

^e^Forward sequence (F).

^f^Reverse sequence (R).

### 2.7. Intestinal Microbiome Analysis by High‐Throughput Sequencing

The 16S rDNA V3–V4 region was amplified, and PCR amplicons were purified with Vazyme VAHTSTM DNA Clean Beads (Vazyme, Nanjing, China), and Quant‐iT PicoGreen dsDNA Assay Kit (Invitrogen, Carlsbad, CA, USA) was used for quantitative analysis of DNA. After the individual quantification step, amplicons were pooled in equal amounts, and pair‐end 2 × 250 bp sequencing was performed using the Illumina NovaSeq platform with NovaSeq 6000 SP Reagent Kit (500 cycles) at Shanghai Personal Biotechnology Co., Ltd. (Shanghai, China).

Sequence data analyses were mainly performed using QIIME2 and R packages (v3.2.0). Amplicon sequence variant (ASV) level ranked abundance curves were generated to compare ASV richness and evenness among samples. The α‐diversity was assessed by Chao1, Shannon, observed species, Faith’s PD, Pielou’s evenness, Good’s coverage and Simpson indices, and visualized as box plots. The β‐diversity was determined to investigate the structural variation of microbial communities across samples using Bray–Curtis metrics and visualized via principal coordinate analysis (PCoA). The significance of differentiation of microbiota structure among groups was assessed by Adonis.

### 2.8. Data Statistics and Analysis

Data were analyzed using SPSS 27.0 software, and graphs were generated with GraphPad Prism 9. The homogeneity of variances was verified using the Levene’s test. For evaluation of significant differences, one‐way analysis of variance (ANOVA) was conducted first, followed by Duncan’s multiple‐range test for post hoc comparisons. Statistical significance was defined at *p* < 0.05, and all data were expressed as means ± standard error of the mean (SEM).

## 3. Results

### 3.1. Growth Performance

As shown in Table 3, dietary BSPC25 resulted in significantly higher final body weight (FBW) and condition factor (CF) than NSPC0 and NSPC25, as well as significantly higher specific growth rate (SGR) and weight gain rate (WGR) than NSPC0 (*p* < 0.05). There were no significant differences in IBW, FCR, DFI, and PER among the three groups.

**Table 3:** Growth performance indices of juvenile hybrid grouper fed with different diets.

Indices	Experimental diets		
	NSPC0	NSPC25	BSPC25
IBW^1^ (g)	8.47 ± 0.31^a^	7.71 ± 0.19^a^	8.30 ± 0.26^a^
FBW^2^ (g)	12.53 ± 0.67^b^	12.15 ± 0.73^b^	14.68 ± 0.28^a^
SGR^3^ (%/day)	0.65 ± 0.07^b^	0.75 ± 0.06^ab^	0.95 ± 0.07^a^
CF^4^ (100 W/L^3^)	1.37 ± 0.04^b^	1.39 ± 0.02^b^	1.59 ± 0.02^a^
WGR^5^ (%)	47.98 ± 6.25^b^	57.26 ± 5.51^ab^	77.40 ± 7.89^a^
FCR^6^	3.37 ± 0.34^a^	3.01 ± 0.33^a^	2.42 ± 0.15^a^
DFI^7^ (%/day)	2.12 ± 0.06^a^	2.19 ± 0.07^a^	2.22 ± 0.04^a^
PER^8^ (%)	62.42 ± 6.90^a^	70.12 ± 7.52^a^	85.58 ± 5.24^a^

*Note:* All values expressed as the mean ± SEM (*n* = 3). Values in the same row with different superscript lowercase letters represent a significant difference (*p* < 0.05). DFI, average daily feed intake.

Abbreviations: CF, condition factor; FBW, final body weight; FCR, feed conversion ratio; IBW, initial body weight; PER, protein efficiency ratio; SGR, specific growth ratio; WGR, weight gain rate.

^1^IBW (g) = initial body weight/initial amount of fish.

^2^FBW (g) = final body weight/final amount of fish.

^3^SGR (%/d) = 100 × (Ln final body weight − Ln initial body weight)/number of days.

^4^CF (100·W/L^3^) = 100 × body weight/(body length)^3^.

^5^WGR (%) = 100 × (final body weight − initial body weight)/initial body weight.

^6^FCR = total feed intake/total weight gain.

^7^DFI (%/d) = 100 × feed intake/[(initial body weight + final body weight)/2 × experimental period].

^8^PER (%) = weight gain/protein intake.

### 3.2. Intestinal Digestive Enzymatic Activity

Figure 1 shows that there were no significant differences in amylase, trypsin, and lipase activities among all groups. However, the BSPC25 group had the trend of higher activities in each enzyme than the NSPC25 group.

Figure 1Effects of three diets on intestinal digestive enzyme activity in juvenile hybrid grouper. (A) Amylase, (B) Typsin, (C) Lipase. All values are expressed as the mean ± SEM (*n* = 3). ns indicates no significant difference between groups in Duncan’s test (*p* > 0.05).

**Figure:**
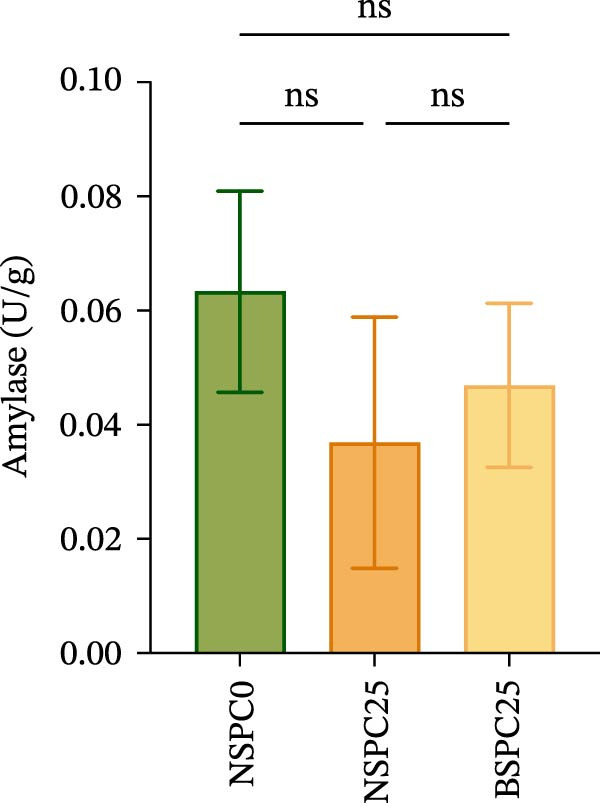
(A)

**Figure:**
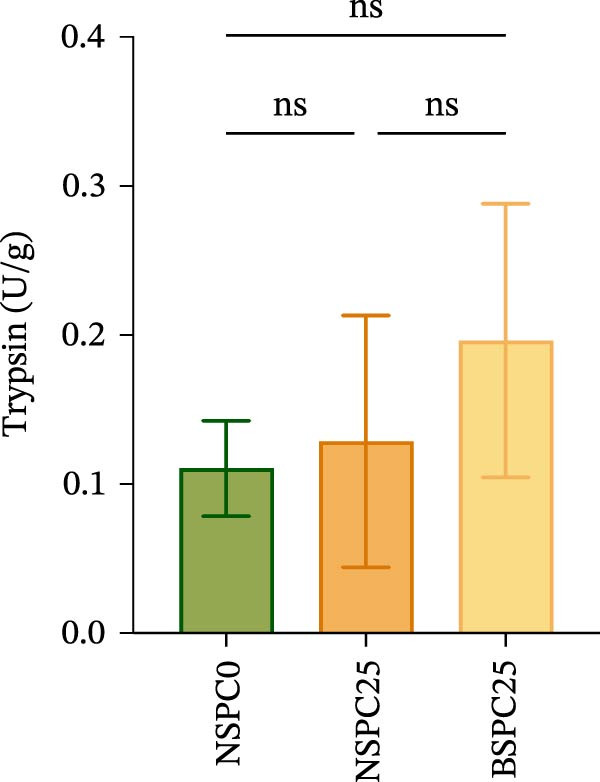
(B)

**Figure:**
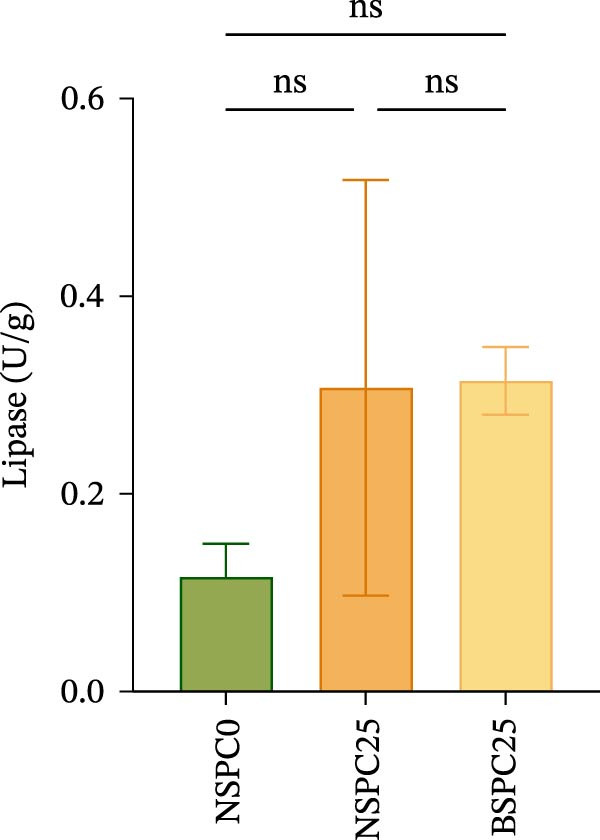
(C)

### 3.3. Hepatic Antioxidant Enzyme Activity

Figure 2 displays the effects of the experimental diets on the liver antioxidant enzyme activity in juvenile hybrid grouper. After 60 days of the feeding trial, there was no significant difference in SOD activity among all groups, but the NSPC25 and BSPC25 groups showed significantly higher activities in MDA than the NSPC0 group.

Figure 2Effects of three diets on liver antioxidant enzyme activity in juvenile hybrid grouper. (A) SOD, (B) MDA. All values are expressed as the mean ± SEM (*n* = 3). ns indicates no significant difference between groups in Duncan’s test (*p* > 0.05), whereas the asterisk ( ^∗^) indicates significant differences between groups in Duncan’s test (*p* < 0.05).

**Figure:**
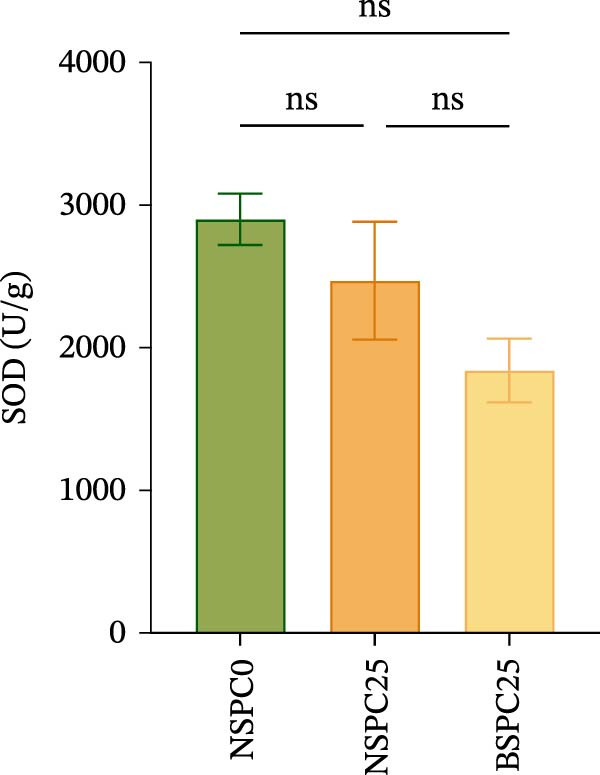
(A)

**Figure:**
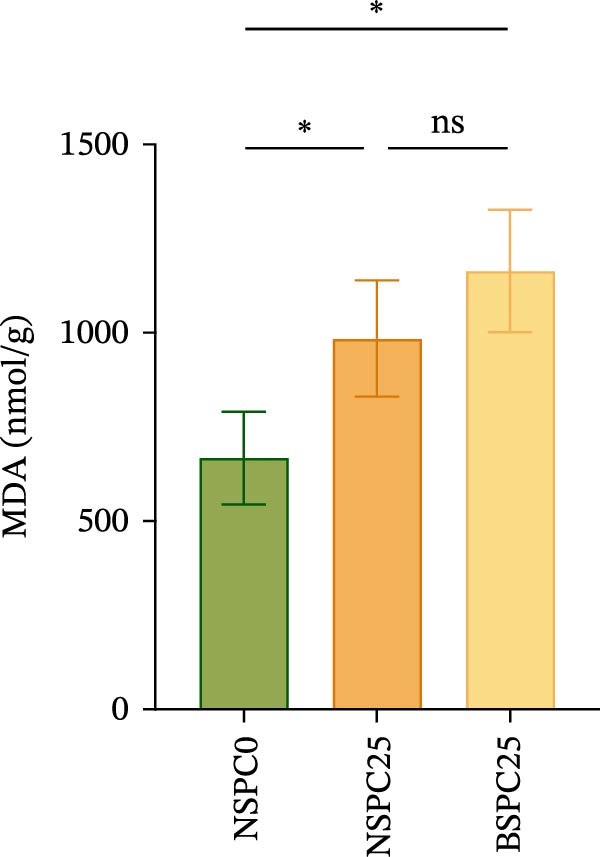
(B)

### 3.4. Real‐Time Quantitative RT‐PCR

Figure 3 shows that the BSPC25 group had the highest hepatic level of TOR (*p* < 0.05). The level was not significantly different from that of the NSPC25 group (*p* > 0.05). There were no significant differences in intestinal PepT1, hepatic IGF‐1, and GH among the three groups (*p* > 0.05).

Figure 3Expression levels of (A) PepT1 in the intestine and (B) IGF‐1, (C) GH, and (D) TOR in the liver. All values are expressed as mean ± SEM (*n* = 3). ns indicates no significant difference between groups in Duncan’s test (*p* > 0.05), whereas the asterisk ( ^∗^) indicates significant differences between groups in Duncan’s test (*p* < 0.05).

**Figure:**
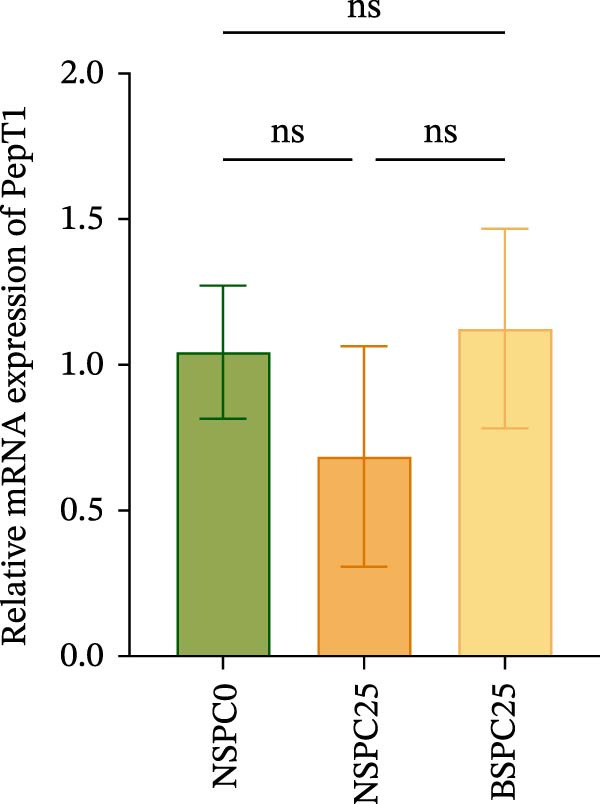
(A)

**Figure:**
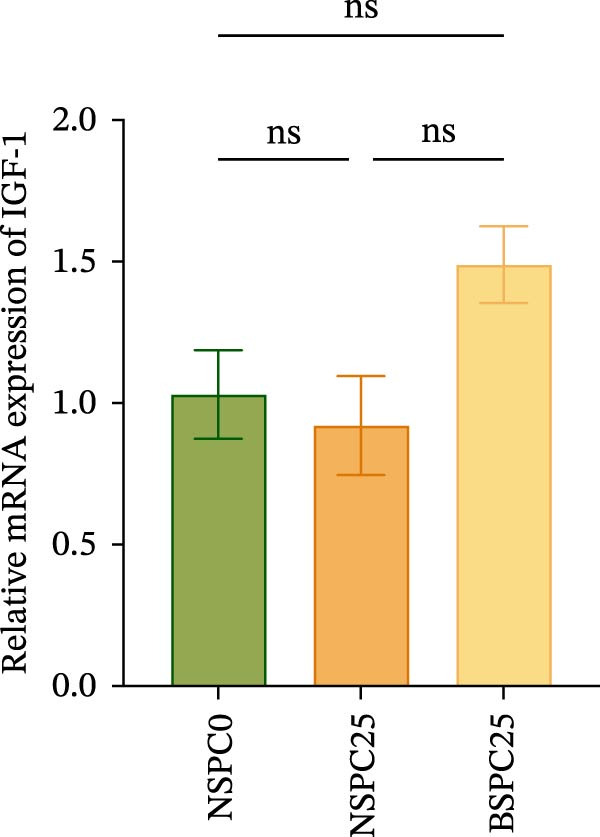
(B)

**Figure:**
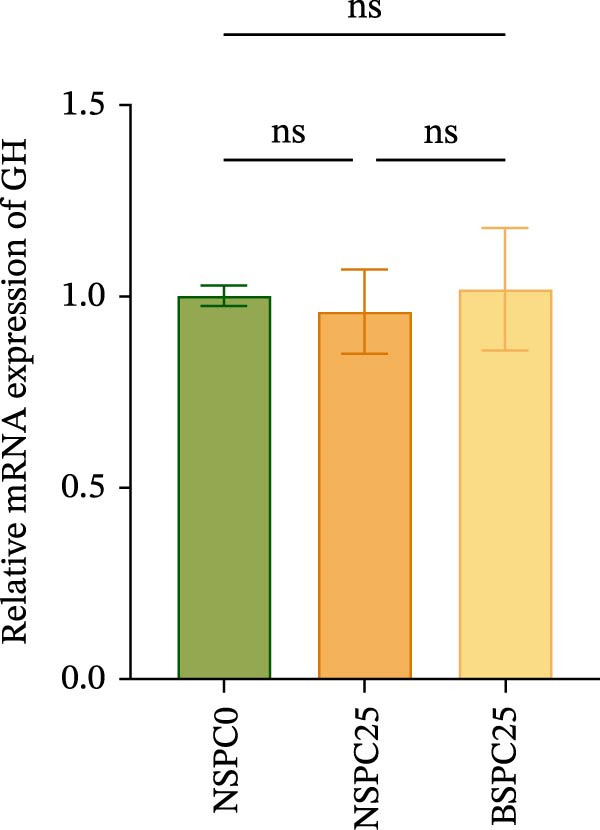
(C)

**Figure:**
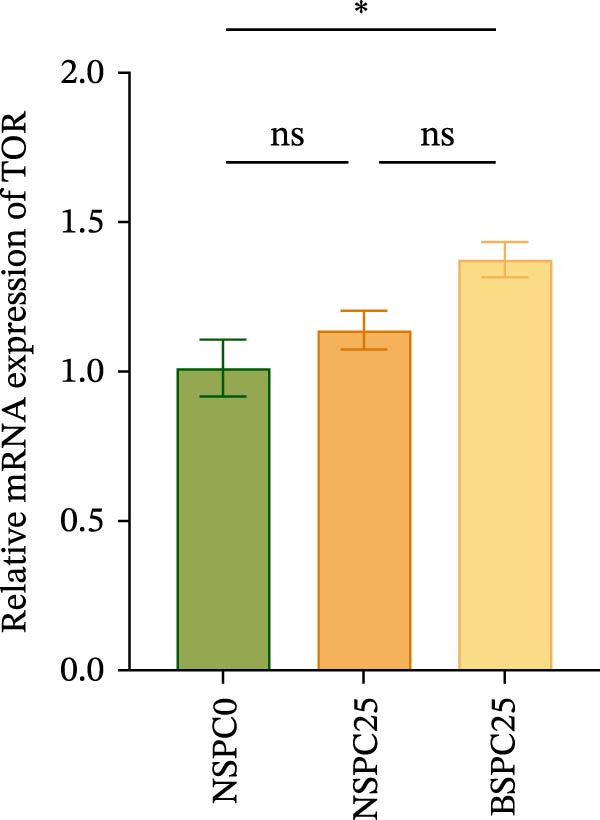
(D)

### 3.5. Analysis of Intestinal Microbiota

**Figure 4:**
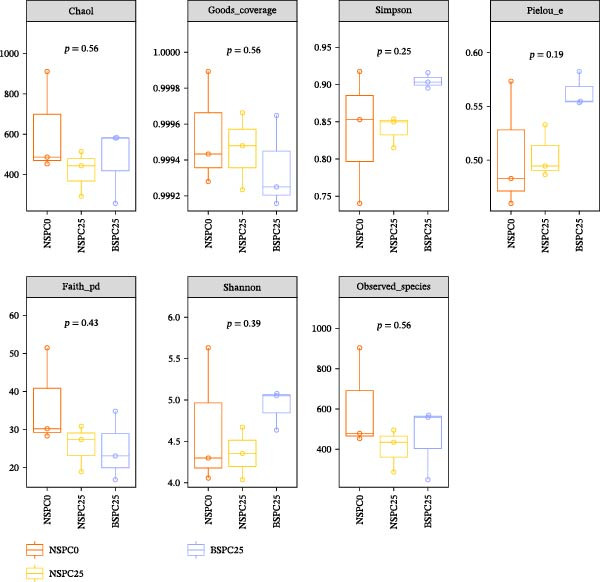
Effects of three diets on intestinal microbial α‐diversity. The statistical test among groups was conducted using Kruskal–Wallis H and Dunn. *p* < 0.05 indicated a significant difference among the three groups.

As shown in Figure 4, there were no significant differences in α‐diversity (*p* > 0.05) among the three groups, including the abundance index Chao1, and the species diversity index Shannon and Simpson, the sequencing coverage Good’s coverage, the phylogenetic diversity Faith’s PD, the species distribution uniformity Pielou’s evenness, and the observed species.

**Figure 5:**
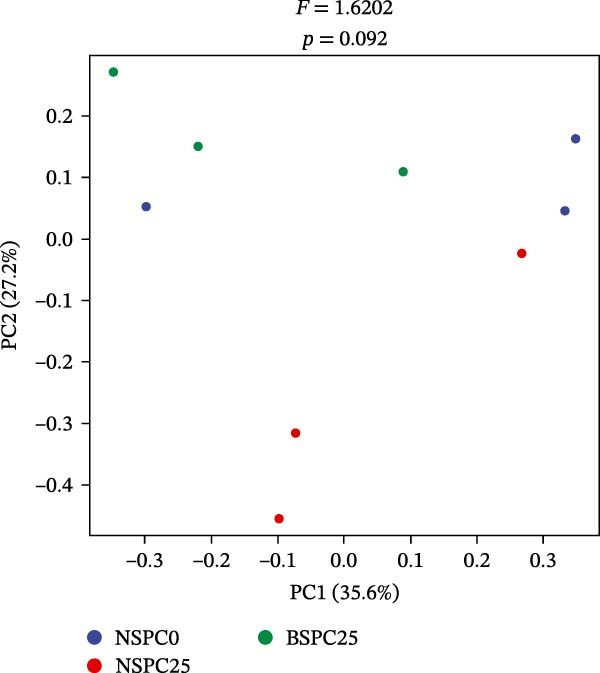
Characterization of intestinal microbial communities with principal coordinate analysis (PCoA) plots of β‐diversity based on a weighted Bray–Curtis metric. The significance of differentiation of microbiota structure among groups was assessed by Adonis.

In PCoA (Figure 5), the results showed that there was no significant aggregation or dispersion among NSPC0, BSPC25, and NSPC25, indicating a high compositional similarity between these samples (*p* > 0.05).

At the phylum level (Figure 6A), Proteobacteria, Bacteroidota, Verrucomicrobiota, and Patescibacteria were the four predominant phyla (average relative abundance > 1%) in the NSPC0 and NSPC25 groups. Proteobacteria and Bacteroidota were dominant phyla in the BSPC25 group. At the genus level (Figure 6B), the prevalent genera in juvenile hybrid grouper intestine comprised *Vibrio*, *Phaeobacter*, *Donghicola*, *Photobacterium*, *Polaribacter_A*, *Aliiroseovarius*, *Acinetobacter*, and *Maritalea*, and there were no significant differences among the three groups.

Figure 6Effects of three diets on the intestinal microbial composition of juvenile hybrid grouper. (A) Microbial relative abundance of intestinal bacterial community at the phylum level and (B) microbial relative abundance of intestinal bacterial community at the genus level. The statistical test was conducted using the Duncan’s test.

**Figure:**
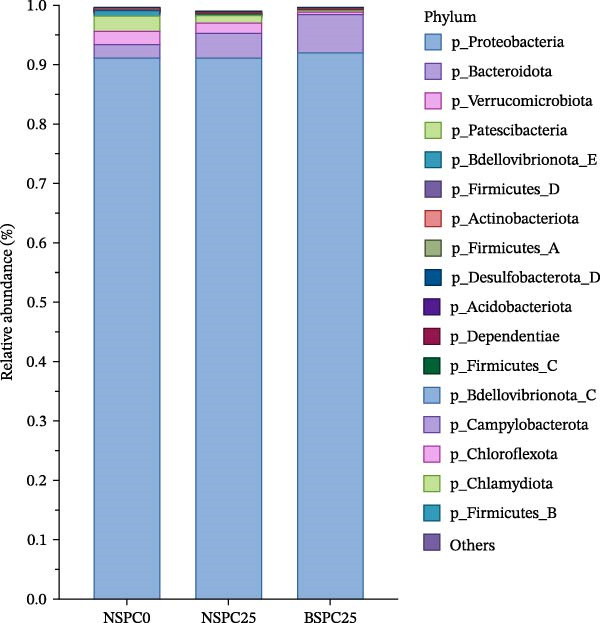
(A)

**Figure:**
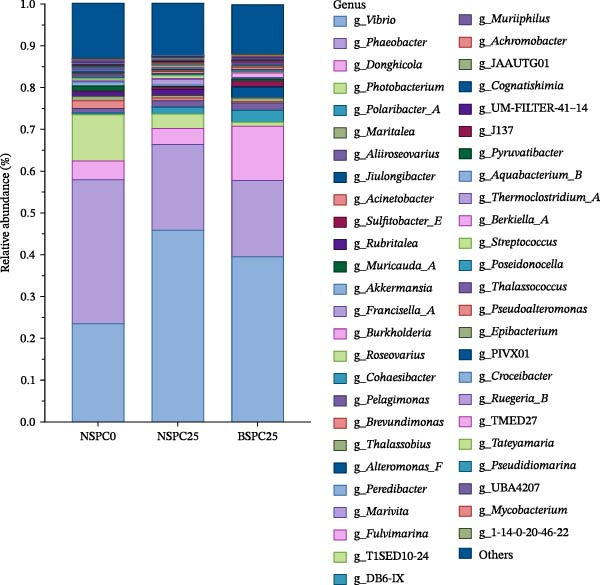
(B)

## 4. Discussion

Due to its nonpathogenic and nontoxic characteristics, *B. coagulans* is widely used as a probiotic additive in feed in aquaculture [16–18]. There have been a growing number of studies focusing on the effects of probiotics in aquafeeds on the growth performance, intestinal microbiota, digestive capacity, immune function, and protein synthesis ability of aquatic organisms when incorporated into various feed formulations [15, 19]. Some studies have investigated the use of *B. coagulans* in some aquatic organisms, livestock, and poultry feeds [11, 20]. However, this is the first report on the application of *B. coagulans* to juvenile hybrid grouper to the best of our knowledge. The study evaluated the effects of three feeds with/without SPC substitution and *B. coagulans* supplementation on the growth performance, digestive enzyme activity, antioxidant capacity, gene expression, and gut microbiota of hybrid grouper.

Many studies have demonstrated that when the substitution proportion of soybean protein for fish meal exceeds certain thresholds, it may negatively affect the growth traits of grouper, damage the intestines, lead to oxidative reactions, cause inflammation, and even reduce its survival rates [21]. Here, our results showed that BSPC25, a SPC substituted diet with *B. coagulans* supplementation, significantly improved the growth performance (FBW, CF, SGR, and WGR) of hybrid grouper (*p* < 0.05), while showed not significantly effect on FCR and DFI (*p* > 0.05). These results indicated that *B. coagulans* can promote hybrid grouper growth by improving the nutrient utilization efficiency rather than simply increasing the feed intake, which is consistent with the conclusions of several studies. For example, the addition of *B. coagulans* to *Litopenaeus vannamei* feed significantly increased its WGR and SGR while significantly reduced its FCR [22]. In *Carassius auratus gibelio*, supplementation with 500 mg/kg *B. coagulans* significantly increased the FBW and PER but notably decreased the FCR [23].

Digestive enzyme activity has important impacts on the feed utilization and growth status of fish [24]. The BSPC25 group had the trend of higher activities of trypsin, amylase, and lipase than the other two groups, though the differences among the three groups were not significant (*p* > 0.05), indicating that *B. coagulans* can improve the digestion of SPC, which has also been found in other studies. In the study of common carp (*Cyprinus carpio* L.), dietary supplementation of *B. coagulans* improved the digestive enzyme activities to varying degrees compared with the control, but not all improvements were significant, which may be related to the applied dose of *B. coagulans* and differences between species [25]. In the study of *Eleutheronema tetradactylum*, *B. coagulans* supplementation significantly reduced the trypsin and amylase activities (*p* < 0.05), but significantly improved the intestinal villus height and growth traits, such as WGR and CF (*p* < 0.05), suggesting that *B. coagulans* effectively promotes its growth performance and gut health [26].

The PepT1, IGF‐1, GH, and TOR genes are the core factors regulating nutritional status, growth performance, and protein synthesis in fish [27, 28]. In this study, compared with that in the NSPC0 group, the expression of TOR in the liver of the BSPC25 group was significantly increased (*p* < 0.05). The expression of TOR provides a basis for the growth and protein synthesis of hybrid grouper. There have been no studies demonstrating the effect of *B. coagulans* on the TOR signaling pathway in hybrid grouper, but there have been studies confirming that probiotics can activate this pathway. In *Danio rerio*, *Lactobacillus casei* upregulated the relative expression of IGF‐1 and GH (*p* < 0.05) [29]. In *Penaeus vannamei*, *B. subtilis* was found to significantly upregulate the TOR gene (*p* < 0.05) [30]. In *Danio rerio*, the expression of IGF‐1 and GH genes was significantly upregulated by low‐dose probiotics, but not significantly changed by high‐dose probiotics (*p* > 0.05), indicating that the expression of these genes is affected by the probiotics in a dose‐dependent manner [31].

SOD and MDA are important indicators of oxidative stress [32]. In this study, the NSPC0 group had a significant difference in MDA compared to NSPC25 and BSPC25 groups, while SOD activity was not significantly different among the three groups and showed a slight decreasing trend in the NSPC25 and BSPC25 groups compared with the NSPC0 group. Surprisingly, there was no significant difference in MDA between NSPC25 and BSPC25 groups, suggesting that *B. coagulans* had no significant adverse immune reaction to 25% SPC substitution for fish meal. A previous study has shown that compared with the high and low fish meal groups, the low fish meal group supplemented with *B. coagulans* had significant increases in SOD activity but no significant difference in MDA activity [15]. Several studies have also shown the high antioxidant capacity of *B. coagulans* both in vitro and in vivo [12, 33–35]. These studies enlighten us that, to further improve the antioxidant capacity of *B. coagulans* on fish, it was necessary to further design a gradient experiment to replace fishmeal with SPC to verify it.

The gut microbiota can regulate the growth of the host, and by changing the gut microbiota and utilizing their own metabolic capacity, bacterial metabolites play an important role in the physiological homeostasis of the host and the intestinal flora [36, 37]. In this study, the NSPC0, NSPC25, and BSPC25 groups showed no significant difference in ASV level ranked abundance curves, α‐diversity, and β‐diversity indices (*p* > 0.05). However, the BSPC25 group reduced the abundance of *Vibrio* and *Photobacterium*, two common harmful pathogens in the intestinal microbiota of hybrid grouper, indicating that *B. coagulans* can regulate the balance of intestinal microbes and maintain intestinal health [38, 39]. Similarly, a previous study showed that supplementation with host gut‐derived *Bacillus* bacteria decreased the relative abundance of *Vibrio* and *Photobacterium* in the gut of hybrid grouper [40]. In *Litopenaeus vannamei*, supplementation with *B. coagulans* also significantly decreased the relative abundance of the *Vibrio* and *Photobacterium* [22]. For the decrease in the relative abundance of *Vibrio* and *Photobacterium*, other studies have achieved clear statistical significance, whereas our results only showed a decreasing trend. This suggests that it is necessary to further improve our experimental protocols (such as modifying the strain concentration and including more gradients for fish meal substitution with SPC) for more effects, so as to study that dietary *B. coagulans* supplementation can promote the gut health of hybrid grouper.

## Funding

This work was supported by the National Natural Science Foundation of China (Grant 32160866).

## Conflicts of Interest

The authors declare no conflicts of interest.

## Supporting Information

Additional supporting information can be found online in the Supporting Information section.

## Supporting information


**Supporting Information** Information on statistical analysis of CT values of the internal reference gene β‐actin.

## Data Availability

The data that support the findings of this study are available from the corresponding authors upon reasonable request.
