# Supplementary material for: Soy Protein Concentrate Dietary Substitution and Bacillus coagulans Supplementation Influence the Growth Performance, Digestive Enzyme Activity, Antioxidant Capacity, Gene Expression, and Gut Microbiota of Hybrid Grouper (Epinephelus fuscoguttatus♀ × Epinephelus lanceolatus♂)
Source: Aquac Nutr. 2026 Apr 22;2026:4176337. doi: 10.1155/anu/4176337 (PMC13100649; doi:10.1155/anu/4176337)
Supplement: Supplementary file 1 — Supporting Information Information on statistical analysis of CT values of the internal reference gene β‐actin. [file ANU-2026-4176337-s001.docx]

Table S1 Statistical analysis of CT values of the internal reference gene β-actin

| Sample | Method | *P* value | | | |
| --- | --- | --- | --- | --- | --- |
|  |  | GH | TOR | IGF-1 | PepT1 |
| NSPC0 | Shapiro-Wilk (Normality) | 0.808 | 0.808 | 0.885 | 0.886 |
| NSPC25 | Shapiro-Wilk (Normality) | 0.663 | 0.663 | 0.032 | 0.587 |
| BSPC25 | Shapiro-Wilk (Normality) | 0.463 | 0.463 | 0.107 | 0.846 |
| NSPC0, NSPC25, and BSPC25 | Levene (Homoscedasticity) | 0.183 | 0.183 | 0.212 | 0.181 |
| NSPC0, NSPC25, and BSPC25 | One-Way ANOVA / Kruskal-Wallis H (Significance) | 0.133 | 0.133 | 0.252 | 0.230 |
